# GalaxySite: ligand-binding-site prediction by using molecular docking

**DOI:** 10.1093/nar/gku321

**Published:** 2014-04-21

**Authors:** Lim Heo, Woong-Hee Shin, Myeong Sup Lee, Chaok Seok

**Affiliations:** 1 Department of Chemistry, Seoul National University, Seoul 151-747, Korea; 2 Department of Biomedical Sciences, University of Ulsan College of Medicine, Seoul 138-736, Korea

## Abstract

Knowledge of ligand-binding sites of proteins provides invaluable information for functional studies, drug design and protein design. Recent progress in ligand-binding-site prediction methods has demonstrated that using information from similar proteins of known structures can improve predictions. The GalaxySite web server, freely accessible at http://galaxy.seoklab.org/site, combines such information with molecular docking for more precise binding-site prediction for non-metal ligands. According to the recent critical assessments of structure prediction methods held in 2010 and 2012, this server was found to be superior or comparable to other state-of-the-art programs in the category of ligand-binding-site prediction. A strong merit of the GalaxySite program is that it provides additional predictions on binding ligands and their binding poses in terms of the optimized 3D coordinates of the protein–ligand complexes, whereas other methods predict only identities of binding-site residues or copy binding geometry from similar proteins. The additional information on the specific binding geometry would be very useful for applications in functional studies and computer-aided drug discovery.

## INTRODUCTION

Proteins perform their biochemical functions by interacting with other biomolecules such as small ligands, other proteins or nucleic acids. The detection of binding site on a protein makes it possible to infer the function of the protein and provides information on binding pockets crucial for computer-aided drug discovery (1,2). Ligand-binding-site predictions from protein sequences have important implications with regard to sequence-based predictions of the functions of proteins. Binding-site prediction on known experimental protein structures is also important when the known structures do not contain ligands or can bind other ligands. Various evolutionary information-based, geometry-based, energy-based and combined methods have been reported (3). 

Recently, methods that use experimental structures of similar protein–ligand complexes have been successfully applied in binding-site predictions in critical assessment of structure prediction (CASP) experiments (4–7). In such methods, binding-site information of homologous proteins of known structures is utilized by assuming that similar protein–ligand contacts occur in the target protein. These methods predict only ligand-binding residues or ligand-binding geometry based on simple structure superimposition to similar protein–ligand complexes (8–12). In this paper, we introduce a new method that uses such information in the context of protein–ligand docking. Because specific binding of ligands to proteins occurs owing to favorable physicochemical interactions, it can be expected that binding-site prediction based on physical chemistry using molecular docking can provide predictions that are more precise. In addition to revealing the identities of the contacting residues, molecular docking can also provide detailed information on atomic interactions between protein and ligand in terms of the optimized 3D coordinates of the protein–ligand complex. The binding geometry obtained by docking can be different from the geometry obtained by simple structure superimposition with similar proteins, and the binding pose optimized by docking tends to have physically more realistic geometry with no severe steric clashes. Such precise information would be very useful for the prediction of specific functions and applications in drug discovery.

However, a few difficulties have to be overcome to apply molecular docking to binding-site prediction methods. First, docking requires prior knowledge of the protein structure and binding ligand. Second, docking results can be sensitive to structural details, and the prediction accuracy may decrease if the protein structure is not sufficiently accurate or if conformational changes occur upon binding (8). In the GalaxySite program, binding ligand is predicted using a similarity-based method, and the protein structure is provided by the user or predicted from a template-based modeling method. The current binding-site prediction method is accurate even when only chemically similar ligands are predicted. The energy function for docking is designed to be less sensitive to structural details by adapting a combination of physics-based terms of AutoDock3 (13) and restraint terms derived from homologous protein–ligand complexes of known experimental structures.

GalaxySite has been tested on the following non-metal ligand-binding-site prediction test sets in addition to the blind prediction test sets of CASP9 and CASP10: 644 nucleotide-binding proteins with known experimental structures, 46 holo/apo pairs of proteins with experimentally resolved structures and 480 targets of the ligand-binding-site prediction category from the continuous automated model evaluation server (CAMEO; http://www.cameo3d.org/lb/) released between 16 August and 8 November 2013. In these tests, the performance of GalaxySite was superior or comparable to other state-of-the-art prediction methods.

## MATERIALS AND METHODS

### Overall procedure

**Figure 1.**
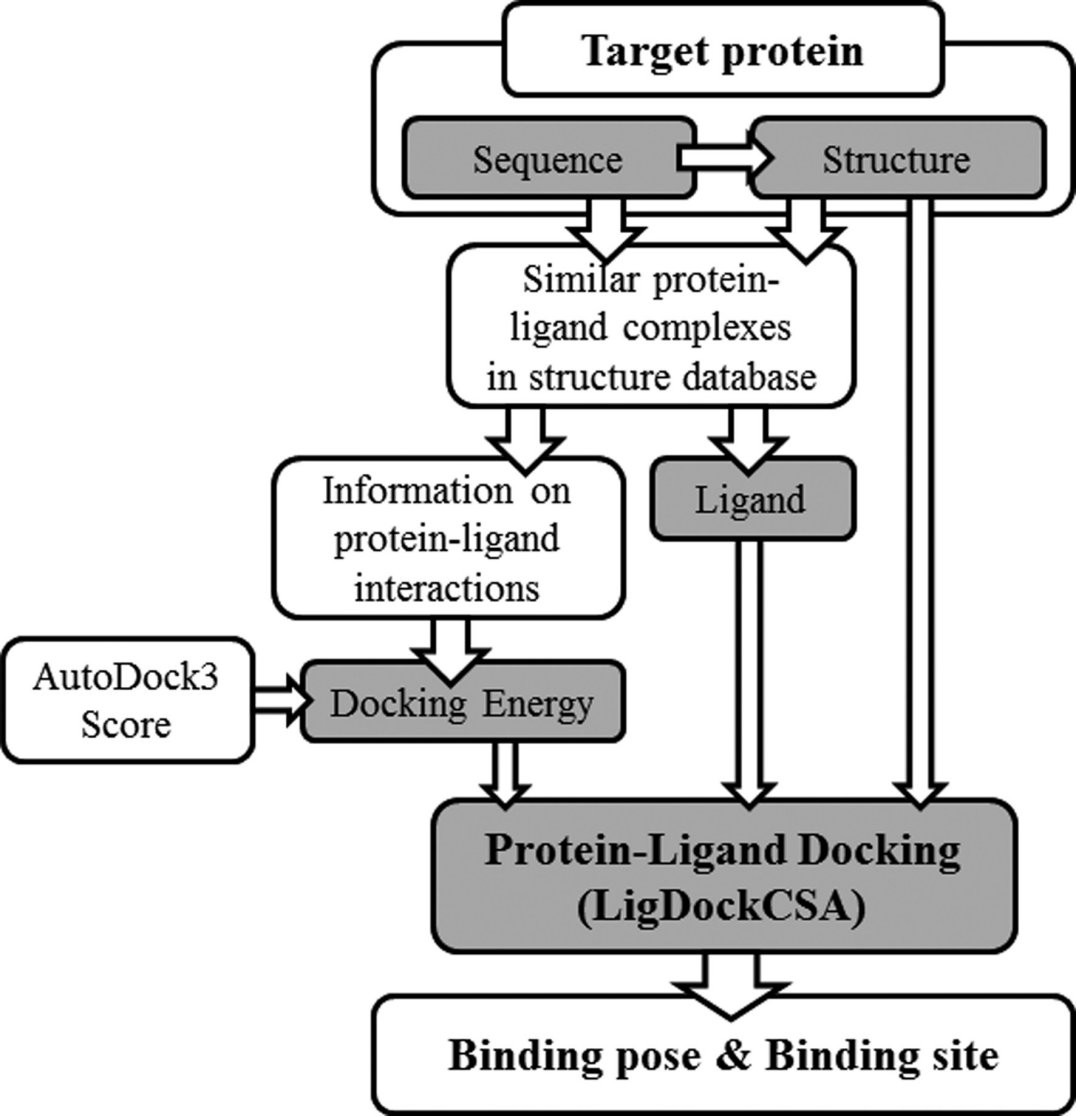
Flowchart of the GalaxySite algorithm. The ligand-binding site of a protein is predicted by protein–ligand docking. The protein structure required for the docking simulation may be provided by the user or predicted from the protein sequence. The binding ligand is predicted from similar protein–ligand complexes in the structure database. The molecular docking algorithm LigDockCSA is used with a hybrid energy of AutoDock3 energy and restraint energy derived from similar protein–ligand complexes. Binding-site residues are extracted from the docking pose.

The GalaxySite program predicts the ligand-binding site of a given protein by protein–ligand docking, as shown schematically in Figure 1. The GalaxySite program uses a protein sequence or structure as input. The input structure may be either an experimental or a predicted structure. If a protein sequence is provided, GalaxySite predicts the protein structure using GalaxyTBM, a template-based modeling method (14,15). Up to three non-metal ligands are extracted from the protein–ligand complex structures of similar proteins detected by HHsearch (16). Ligand-binding poses are then predicted using LigDockCSA (17).

### Prediction of binding ligands

Ligands to be docked to the target protein structure are predicted from experimental structures of template proteins with bound ligands. The template search is performed in the protein structure database ‘pdb70’ with a maximum mutual sequence identity of 70% via HHsearch (16) in the local alignment mode. Out of the 30 proteins with the highest re-ranking score calculated from the HHsearch results (14,15), proteins whose structures are very different from that of the target protein are filtered out, and the remaining proteins are selected as templates. The criterion used for filtering out dissimilar structures depends on the similarity of the target protein structure to the closest template among the top 30 proteins. Structures with TM-score [similarity to the target structure calculated using TM-align (18)] <0.5, <0.4 and <0.3 were filtered out when TM-score of the closest template is >0.8, >0.6 and ≤0.6, respectively. In this way, prediction accuracy is enhanced with the use of stricter criterion [for example, TM-score >0.5 to include only those proteins that share the same fold (19)] when more accurate prediction is expected (for example, when the similarity of the best template to the target structure is very high with TM-score >0.8), and prediction coverage is enhanced with less strict criterion when less accurate prediction is expected. Among the non-metal ligands bound to the templates, non-biological ligands such as sulphate ion, glycerol and polyethylene glycol that are added to facilitate crystallization are filtered out first. See Supplementary Information for a complete list of the ligands considered non-biological. Ligands with high positional variation (>10 Å) of the center atoms in superposed template structures that contain the same ligand are also filtered out. The remaining ligands are ranked according to the sum of the HHsearch re-ranking score of templates that contain the same ligand; up to three ligands with the highest rank are used in the docking calculations. The overall procedure of binding-ligand selection was trained on the CASP7 function prediction targets.

### Molecular docking

GalaxySite uses the LigDockCSA (17) protein–ligand docking program that performs global optimization by using the conformational space annealing (CSA) algorithm (17, 20–22). The protein structure is fixed at the initial input or model structure, and the ligand is considered fully flexible. A pool of 100 conformations is first generated by perturbing the initial conformations obtained from template ligand poses. The pool is then evolved by generating trial conformations and comparing the trial conformations with the pool conformations, gradually focusing on narrower regions of lower energy in the conformational space. Details on the docking algorithm can be found elsewhere (17). Out of the final pool of 100 structures, the pose with the lowest docking energy in the largest cluster is selected as representative binding pose.

The energy function used for docking is expressed as follows:

(1) \begin{equation*} E = E_{{\rm AutoDock}} + 1.1E_{{\rm Restraint}} ,
                            \end{equation*}

where *E*_AutoDock_ is the same as the AutoDock3 energy function (13) except that the maximum energy value for each interacting atom pair is set to 1.0 kcal/mol to tolerate steric clashes that may be caused by inaccurate protein model structures or ligand-unbound structures. The restraint term *E*_Restraint_ is derived from the template structures that contain the selected ligand. Restraint is applied to each ligand atom *i*, imposing a penalty on *r_ij_* (the distance between ligand atom *i* and protein atom *j*) deviating from *r_ij_*^(*k*)^ (the corresponding distance in the *k*th template) with template-dependent weight factor *ω_ijk_*, and the total restraint energy is expressed as follows:

(2) \begin{eqnarray*} &&E_{{\rm Restraint}} (\{ r_{ij}
                            \} ) = \nonumber \\ &&- \sum\limits_i {\ln \left[
                            {\sum\limits_j {\sum\limits_k {\omega _{ijk} \exp { \{ - ( {{r_{ij} -
                            r_{ij}^{(k)} })^2/{d_{jk}^2 }} } \}} } } \right]} ,
                            \end{eqnarray*}

where *d_jk_* is the position deviation of the C_α_ atom of the residue to which the *j*th atom belongs in the target structure from that in the *k*th template when target and template structures are superimposed. The weight factor is expressed as

(3) \begin{eqnarray*}
                            &&\omega _{ijk} = \nonumber \\ &&({\rm
                            TM-score})_k ({\rm Residue score})_{jk} {E_{{\rm AutoDock},ij}
                            (r_{ij}^{(k)} )}/ \nonumber \\ &&{E_{{\rm AutoDock},ij}
                            (r_{\min } )},
                    \end{eqnarray*}

where (TM-score)*_k_* is the structural similarity between the *k*th template and the input structure. The second term, (Residue score)*_jk_*, is 0 if the corresponding template residue is not of the same amino acid type as the target residue or if *d_jk_* > 2 Å. Otherwise, the residue score represents side-chain orientation similarity calculated using the dot product of the normalized vectors connecting C_α_ atoms and the side-chain centroid (of the residue to which the *j*th atom belongs) for the input and *k*th template structures. The third term accounts for the optimality of the template distance estimated by the ratio of the AutoDock3 energy value at that distance to the optimal energy. The relative weight of the AutoDock3 energy to the restraint term in Equation (1) is set to 1.1, which produces optimal results for the targets in the CASP7 function prediction category (6).

### Performance of the method

**Figure 2.**
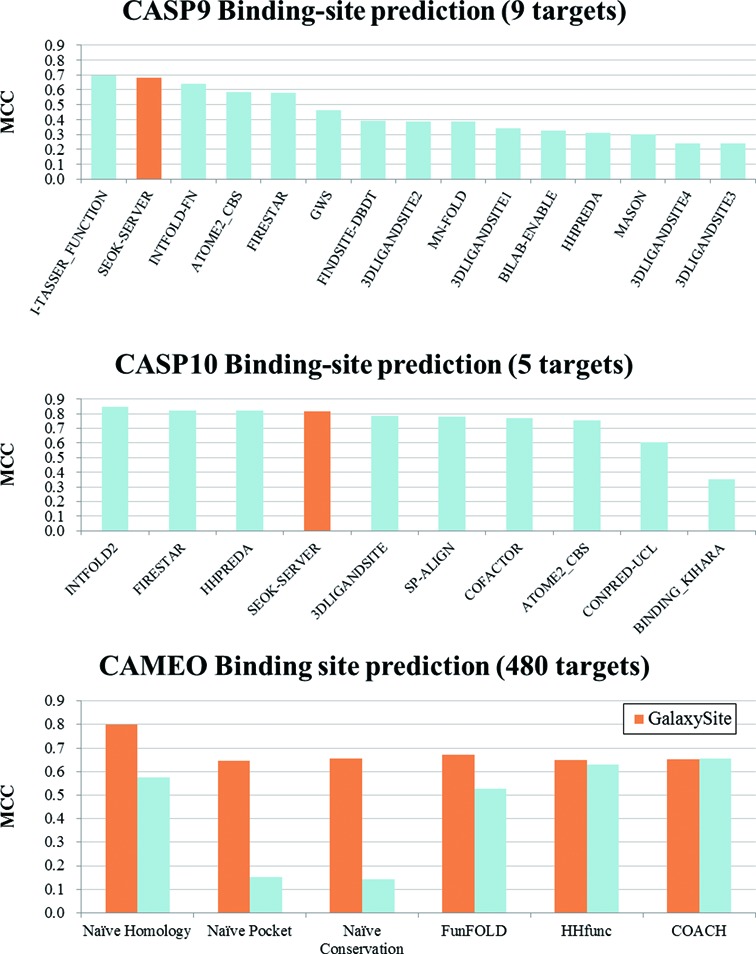
Performance comparison of GalaxySite with other server methods in terms of median Matthews correlation coefficient (MCC) on the CASP9 (top), CASP10 (middle) and CAMEO (bottom) ligand-binding-site prediction category targets. In the figure, the SEOK-SERVER method used GalaxySite in the CASP blind prediction.

GalaxySite has been extensively tested on various types of binding-site prediction test sets. See Supplementary Information for details on the test results. Tests on 644 nucleotide-derived ligand-binding proteins (23) and 46 holo/apo pairs of experimentally resolved structures (24) show that GalaxySite performs superior or comparable to other state-of-art methods in predicting binding sites from protein structures. Prediction from protein sequences was performed on targets in the binding-site prediction category of CASP9 (7) and CASP10 (4) in a blind fashion and on 480 targets from the continuous automated model evaluation server (CAMEO) released between 16 August and 8 November 2013. The prediction accuracy was comparable to other state-of-the-art prediction methods in terms of the Matthews correlation coefficient (MCC) for ligand-contacting residues. See Figure 2 for comparison with other server methods and Supplementary Information for details. It should be noted that the accuracy measures for comparison with other methods depend on the available information provided by other methods and that more detailed, valuable information on specific protein–ligand interactions is available using GalaxySite.

## THE GALAXYSITE SERVER

### Hardware and software

The GalaxySite server runs on a cluster of seven Linux servers of 2.33-GHz Intel Xeon 8-core processors. The web application uses Python and the MySQL database. The ligand-binding-site prediction pipeline is implemented using Python. Open Babel 2.2.3 is used to prepare the ligands for the molecular docking procedure. The molecular docking algorithm for binding-site prediction is implemented in the GALAXY program package (14,15,17,25–28) written in Fortran 90. When a sequence is given as an input, the structure is predicted by GalaxyTBM (14,15) with no additional model refinement. The Jmol software is used for visualization of predicted results.

### Input and output

**Figure 3.**
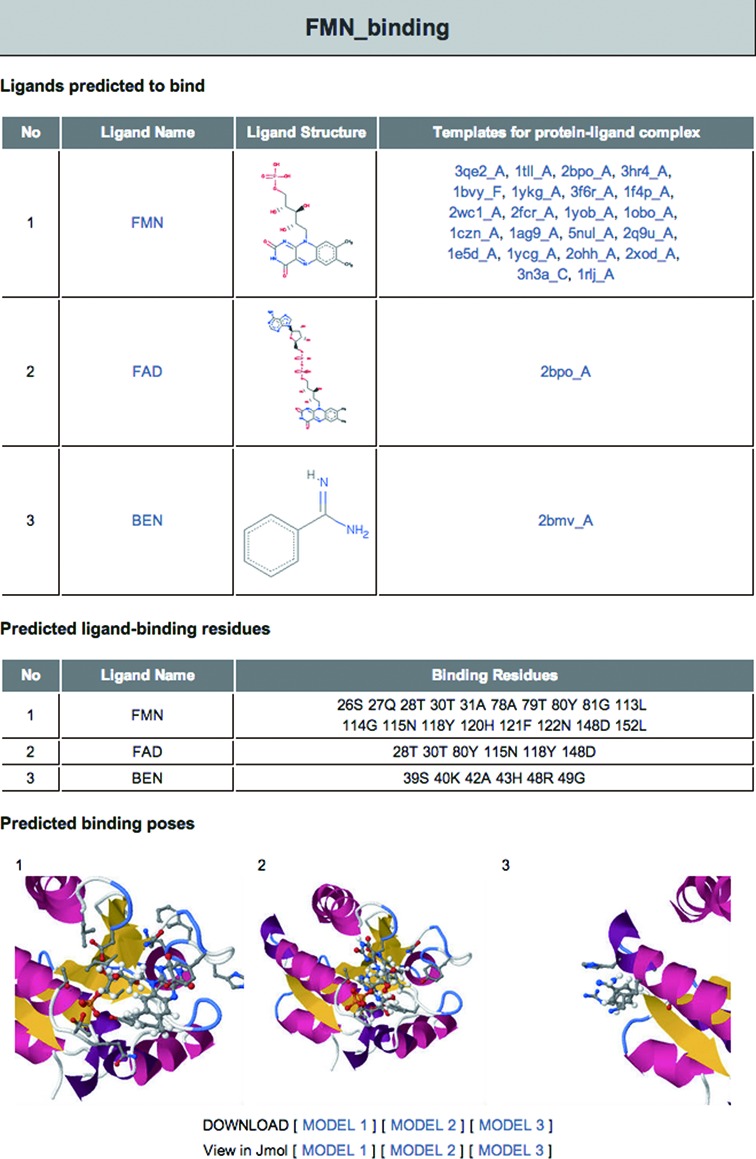
GalaxySite output page. Predicted ligands, their two-dimensional structures and templates for protein–ligand complexes are tabulated. Ligand names and PDB IDs are linked to the RCSB PDB website for detailed information. Predicted ligand-binding residues for each ligand are also listed. Predicted binding poses are shown in static images, which can be viewed using the Jmol structure viewer or can be downloaded in PDB format.

The required input is a protein sequence in FASTA format or a protein structure in PDB format. The number of residues in the input file is limited to 500 for computational efficiency. The average run times are 2 h for a structure input and 4 h for a sequence input. Predictions for up to three non-metal ligands and their template complexes are provided with links to the RCSB PDB website. For each predicted ligand, predicted binding pose and ligand-binding residues can be viewed and downloaded from the website (Figure 3).

## CONCLUSIONS

GalaxySite is a web server for the prediction of binding sites of non-metal ligands that employs molecular docking. The method is applicable to experimentally resolved structures, model protein structures and protein sequences. In addition to information on binding residues provided by previous binding-site prediction methods, GalaxySite predicts specific binding ligands and binding poses that can be useful for further applications, e.g. in computer-aided drug discovery.

## SUPPLEMENTARY DATA

Supplementary Data are available at NAR Online, including [1–7].

## FUNDING

National Research Foundation of Korea (2013R1A2A1A09012229, 2012M3C1A6035362 and 2013R1A1A2058447); Asan Institute for Life Sciences, Seoul, Korea (2013–591 and 2014–607). Funding for open access charge: Seoul National University.

*Conflict of interest statement*. None declared.

## Supplementary Material

Supplementary Data
