# Supplementary material for: GalaxySite: ligand-binding-site prediction by using molecular docking
Source: Nucleic Acids Res. 2014 Apr 21;42(Web Server issue):W210–4. doi: 10.1093/nar/gku321 (PMC4086128; doi:10.1093/nar/gku321)
Supplement: Supplementary Data [file supp_42_W1_W210__index.html]

Supplementary Data 

# GalaxySite: ligand-binding-site prediction by using molecular docking

## Supplementary Data

**Files in this Data Supplement:**

- SUPPLEMENTARY DATA
