# Supplementary material for: GalaxySite: ligand-binding-site prediction by using molecular docking
Source: Nucleic Acids Res. 2014 Apr 21;42(Web Server issue):W210–4. doi: 10.1093/nar/gku321 (PMC4086128; doi:10.1093/nar/gku321)
Supplement: Supplementary Data [file supp_gku321_nar-00390-web-b-2014-File005.doc]

**Supplementary Information**

**GalaxySite: Ligand-binding site prediction
by using molecular docking**

Lim Heo^1^, Woong-Hee Shin^1^, Myeong Sup Lee^2*^ and Chaok Seok1[[1]](#footnote-2)*

*^1^Department of Chemistry, Seoul National University, Seoul 151-747, Korea*

*^2^Department of Biomedical Sciences, University of Ulsan College of Medicine,
Seoul 138-736, Korea*

**Test results for the nucleotide set**

The ‘nucleotide set’ from Kasahara *et al.* (1) consisting of 644 nucleotide-derived ligand-protein complexes was used to validate the method, especially to verify the effectiveness of docking compared to an ‘ideal mapping’ method that copies the ligand pose from the ‘best’ template structure after superimposition of template and query protein structures. The protein structure database ‘pdb70’ released on 10 April 2010 was used, and templates with >70% sequence identity to the target protein were discarded. **Figure S1** compares the results of GalaxySite with ideal mapping in terms of minimum distance (closest distance between any ligand atom in the predicted and native poses) and centroid distance (distance between the centroids of predicted and native poses) for individual targets. The values for average minimum distance and average centroid distance calculated by GalaxySite (1.2 Å and 2.8 Å, respectively) were smaller than those obtained with ideal mapping were (1.4 Å and 3.1 Å, respectively). Considering that the ‘best’ template, i.e., the template with the highest structure similarity to the native complex, is not known in advance in real prediction experiments, this result implies that the docking strategy of GalaxySite is fairly successful in predicting a binding ligand and its docking pose.

Although the exact ligands are predicted only in 52% of the targets, the success rate of predicting ‘similar’ ligands is 80%. [Ligands are considered to be similar if they are in the same group listed as follows: (AMP, ADP, ANP, and ATP); (GDP, GNP, and GTP); and (NAD and NAP).] This suggests that detecting similar ligands can be sufficient to identify the correct binding sites. We found that the success rate of binding-site prediction is more strongly correlated with the identification of similar ligands rather than the identification of the exact ligand, with Pearson’s correlation coefficients of 0.78 and 0.21, respectively.

## Test results for the bound/unbound set

Performance comparison of different binding-site prediction methods is available for the bound/unbound set from Huang and Schroeder (2); it consists of 46 pairs of ligand-bound/ligand-free protein structures. The protein structure database ‘pdb70’ released on 10 April 2010 was used, and templates with >70% sequence identity to the target protein were discarded. The results of this test show the applicability of GalaxySite to binding-site prediction of the protein structure in the ligand-unbound form. As shown in **Table S1**, success rates of GalaxySite were 92% (bound) and 90% (unbound) when the ‘top’ ligand was docked, and 100% and 98% when the ‘top three’ ligands were considered; prediction was considered a success if the shortest distance between the predicted binding pocket centre and any ligand atom in the native structure is <4 Å, which is the same success criterion as described in Huang and Schroeder (2). This performance is comparable or superior to other methods compared in **Table S1**. It is worth emphasizing again that GalaxySite can provide substantially detailed information on ligand binding. It is interesting to note that GalaxySite is not very sensitive to inaccuracies in the input protein structure, showing only 2% decrease in the success rate when an unbound protein structure is used for docking.

The accuracy of ligand prediction is only 26% (‘top’ ligand) and 34% (‘top three’ ligands) for the bound/unbound set. The prediction of chemically similar ligands also contributes to the higher success rate for binding site prediction than the rate of exact ligand prediction. Notably, the ‘top three’ ligands covered the actual ligand type in most of the cases in this test set, resulting in a success rate of 100%.

## Binding site prediction from sequences for CASP targets

Here, we further show that the docking method of GalaxySite is sufficiently effective to be applied to predictions from protein sequences when experimental structures are not available. GalaxySite has been tested on CASP9 (3) and CASP10 (4) in a blind fashion. Predictions on CASP9 targets were performed under group names ‘Seok-server’ for server targets and ‘Seok’ for human/server targets. The prediction methods for ‘Seok-server’ and ‘Seok’ are identical, except that model structures of ‘Seok-server’ and ‘Seok’ were used, respectively.

In **Tables S2** and **S3**, the results of CASP9 and CASP10 are summarized using the three contact-based measures, accuracy, coverage, and Matthews correlation coefficient (MCC) of contacting residues. Distance measures cannot be compared because only the predictions of contact residues are provided in the CASP experiments. Only the results for template-based modelling targets with non-metallic ligands are presented here (9 for CASP9 and 5 for CASP10). We also made predictions for metal-containing proteins in CASP, but the results are not included here because a different prediction strategy was used. Unlike the official CASP9 assessment in which several targets lacking ligands in the experimental structures were included as putative targets, we only considered biologically relevant ligands that were observed in experimental structures, as in the CASP8 official assessment. ‘Seok-server’ and ‘Seok’ that use GalaxySite outperformed other methods, except ‘ZHANG’ and ‘I-TASSER_FUNCTION’ (which are from the same group), with regard to CASP9 targets. For CASP10 targets, the ranks of median and average MCC were very different, probably because of the small number of targets; the ‘Seok-server’ ranks among top servers in terms of median MCC measure. These results suggest that GalaxySite is one of the best available binding-site prediction methods that can be successfully applied to real binding-site predictions from protein sequences.

However, for a few reasons, the above results have to be interpreted with caution. First, the number of test cases is not large enough to draw statistically meaningful conclusions. Second, assessment results may vary depending on the definition of binding-site residues, for example, whether putative binding-site residues showing strong evolutionary conservation are treated as positive, negative, or neutral.

## Binding site prediction from sequences of CAMEO targets

We tested GalaxySite on 480 targets of the ligand-binding site prediction category from the continuous automated model evaluation server released between August 16 and November 8, 2013 (5). The protein structure database ‘pdb70’ released on 12 July 2012 is used. In **Table S4**, results of GalaxySite for these targets were compared with available results of other servers in terms of contact-based measures. In CAMEO, a confidence score is reported for each residue rather than the two-state assignment in CASP. To calculate contact-based accuracy measures, residues with a confidence score of >0.95 were considered to be contacting ligands. Small changes in the cut-off value of the confidence score did not affect the values of accuracy measures a lot. A confidence score for GalaxySite could be developed using the distances to ligand atoms or energy contributions, which can be obtained by docking calculations; however, further investigations in this direction were not carried out. The overall results show that the performance of GalaxySite is consistently comparable or superior to other available server methods for a larger set of prediction targets.

**List of ligands considered non-biological in GalaxySite**

HOH, PO3, BME, IMD, FMT, UNL, FES, EOH, CAS, OH, ACY, TRS, MPD, IOD, MLI, MLT, DTT, ACT, MSE, GOL, EDO, PEG, SO4, CL, ABA, TPO, SEP, UNX, MAN, NAG, NDG, NLE, YCM, DAL, IPA, HEZ, BU1, PG4, P6G, BR, OCS, CSO, MLY, PGE, PE4, 2PE, MES, KCX


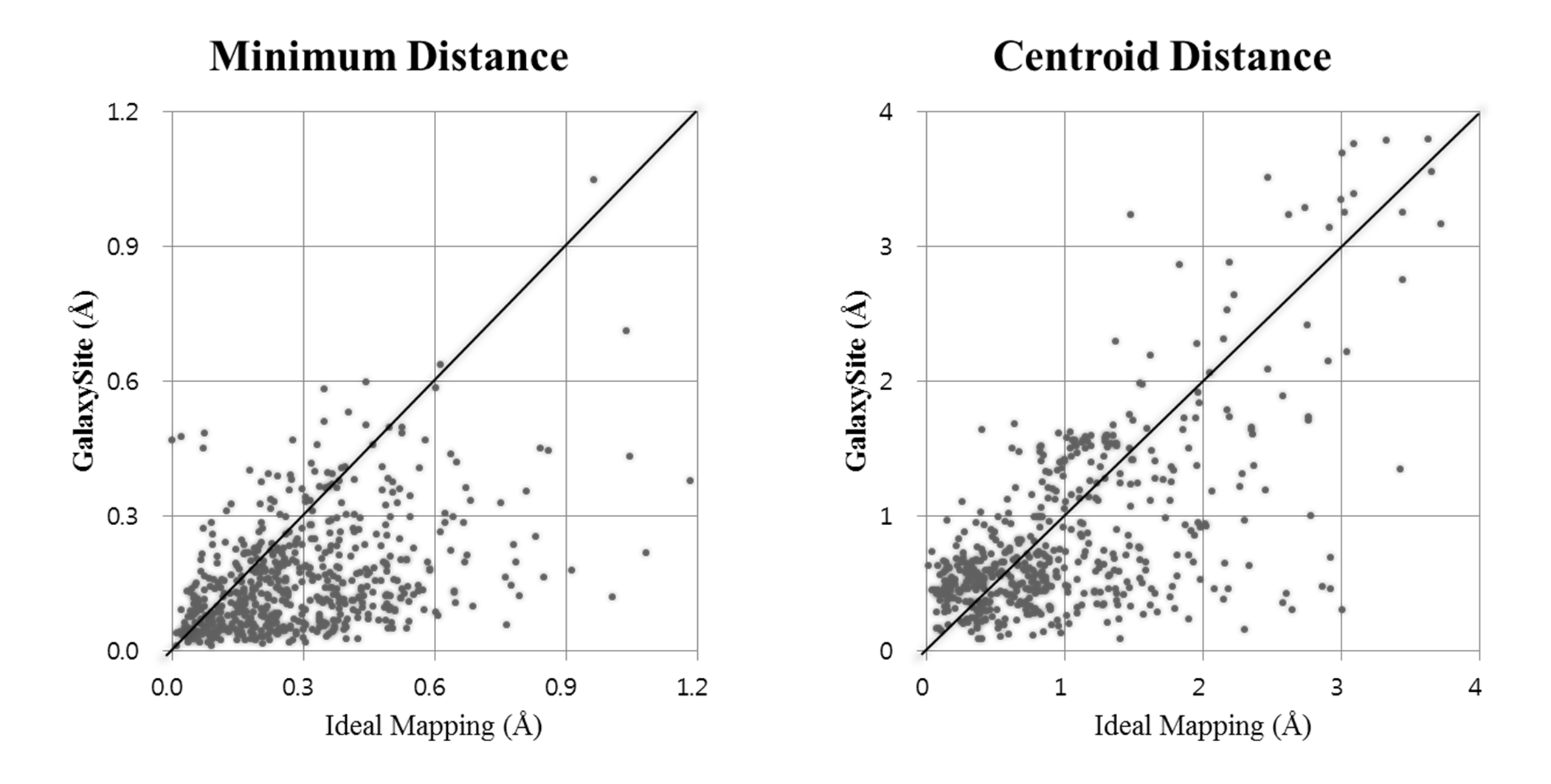


Figure S1. Performance comparison between GalaxySite and ideal mapping for individual targets in the nucleotide set. Ideal mapping refers to mapping the ligand pose of the ‘best’ template selected by structure superimposition onto the native protein structure. GalaxySite shows improved performance both in terms of minimum distance measure (left) and centroid distance measure (right), providing higher-resolution prediction as a result of docking.

**Table S1.** Comparison of success rates of different binding-site prediction methods on the bound/unbound set using the best (top 1) and the best out of the top 3 predictions

| **Method^1)^** | **Top 1** | | **Top 3** | |
| --- | --- | --- | --- | --- |
|  | **Bound** | **Unbound** | **Bound** | **Unbound** |
| **GalaxySite** | **92** | **90** | **100** | **98** |
| FTSITE | - | 94 | - | 98 |
| FINDSITE | 90 | 90 | 94 | 94 |
| VICE | 85 | 83 | 94 | 90 |
| DoGSite | 83 | 71 | 92 | 92 |
| Fpocket | 83 | 69 | 94 | 92 |
| PocketPicker | 72 | 69 | 85 | 85 |
| LIGSITE | 69 | 58 | 87 | 75 |
| CAST | 67 | 58 | 83 | 75 |
| PASS | 63 | 60 | 81 | 71 |
| SURFNET | 54 | 52 | 78 | 75 |

1. Results of GalaxySite and FINDSITE were obtained from our own calculations; those of other methods were taken from (6), except those of FTSITE (7).

**Table S2. Comparison of different binding-site prediction methods on CASP9 binding-site prediction targets with non-metal ligands in terms of median values (average in parentheses) of MCC, accuracy, and coverage**

| **Predictor** | **No. of Predictions**^1)^ | **MCC**^2)^ | **Accuracy**^2)^ | **Coverage**^2)^ |
| --- | --- | --- | --- | --- |
| ZHANG | 8 | 0.710 (0.671) | 72 (63) | 81 (77) |
| SEOK | 7 | 0.709 (0.620) | 76 (65) | 67 (62) |
| I-TASSER_FUNCTION^3)^ | 9 | 0.697 (0.665) | 68 (66) | 80 (74) |
| SEOK-SERVER^3)^ | 8 | 0.682 (0.623) | 81 (69) | 67 (60) |
| INTFOLD-FN^3)^ | 8 | 0.640 (0.570) | 82 (69) | 58 (52) |
| JONES-UCL | 8 | 0.585 (0.602) | 63 (63) | 62 (62) |
| ATOME2_CBS^3)^ | 6 | 0.584 (0.558) | 44 (43) | 87 (82) |
| FIRESTAR^3)^ | 8 | 0.581 (0.562) | 66 (58) | 58 (61) |
| MCGUFFIN | 8 | 0.575 (0.559) | 69 (61) | 62 (56) |
| FAMSSEC | 9 | 0.523 (0.568) | 40 (49) | 75 (75) |
| STERNBERG | 9 | 0.520 (0.513) | 65 (51) | 60 (58) |
| CNIO-FIRESTAR | 7 | 0.508 (0.559) | 67 (58) | 50 (59) |
| LEE | 8 | 0.476 (0.506) | 51 (57) | 51 (53) |
| GWS^3)^ | 9 | 0.464 (0.432) | 50 (54) | 37 (41) |
| KIHARALAB | 9 | 0.431 (0.501) | 50 (51) | 50 (55) |
| LOVELL_GROUP | 9 | 0.412 (0.363) | 40 (44) | 43 (36) |
| TASSER | 9 | 0.396 (0.414) | 41 (40) | 58 (52) |
| FINDSITE-DBDT^3)^ | 8 | 0.394 (0.346) | 35 (34) | 46 (45) |
| 3DLIGANDSITE2^3)^ | 9 | 0.388 (0.343) | 38 (38) | 38 (41) |
| MN-FOLD^3)^ | 9 | 0.387 (0.373) | 24 (27) | 62 (68) |
| 3DLIGANDSITE1^3)^ | 9 | 0.342 (0.346) | 38 (37) | 38 (41) |
| BILAB-ENABLE^3)^ | 9 | 0.326 (0.326) | 21 (29) | 40 (49) |
| HHPREDA^3)^ | 9 | 0.310 (0.291) | 50 (52) | 16 (22) |
| MASON^3)^ | 9 | 0.299 (0.284) | 29 (31) | 36 (33) |
| 3DLIGANDSITE4^3)^ | 9 | 0.239 (0.293) | 19 (30) | 33 (38) |
| 3DLIGANDSITE3^3)^ | 9 | 0.239 (0.284) | 19 (31) | 33 (35) |
| SAMUDRALA | 8 | 0.227 (0.317) | 30 (38) | 29 (33) |

1) Targets: T0516, T0533, T0547, T0597, T0604, T0609, T0632, T0636, T0641

1. Contact-based measures: MCC, accuracy, and coverage. A residue is considered contacting ligand if the distance between any atom in the residue and any ligand atom is less than the sum of the van der Waals radii plus 0.5 Å. By comparing the lists of contact residues in the predicted and the native structures, the three measures are calculated as follows:

$\text{Accuracy}=\frac{TP}{TP+FP}$, $\text{Coverage}=\frac{TP}{TP+FN}$, $\text{MCC}=\frac{TP\times TN-FP\times FN}{\sqrt{(TP+FP)\times(TP+FN)\times(TN+FP)\times(TN+FN)}}$, where *TP*, *TN*, *FP*, and *FN* denote the number of true-positive, true-negative, false-positive, and false-negative predictions for contact residues, respectively. MCC considers both accuracy and coverage, and it is not biased in favour of only one of the two measures.

1. Server predictors

Table S3. Comparison of different binding-site prediction methods on the CASP10 binding-site prediction targets with non-metal ligands in terms of median values (average in parentheses) of MCC, accuracy, and coverage

| **Predictor** | **No. of predictions**^1)^ | **MCC** | **Accuracy** | **Coverage** |
| --- | --- | --- | --- | --- |
| MCGUFFIN | 5 | 0.850 (0.792) | 88 (88) | 82 (75) |
| INTFOLD2^2)^ | 5 | 0.845 (0.802) | 86 (85) | 82 (79) |
| FIRESTAR^2)^ | 5 | 0.821 (0.792) | 80 (81) | 79 (81) |
| HHPREDA^2)^ | 5 | 0.821 (0.752) | 80 (77) | 79 (77) |
| SEOK | 5 | 0.814 (0.750) | 94 (85) | 71 (69) |
| SEOK-SERVER^2)^ | 5 | 0.814 (0.723) | 94 (81) | 73 (68) |
| CNIO | 5 | 0.790 (0.787) | 74 (75) | 87 (87) |
| 3DLIGANDSITE^2)^ | 4 | 0.785 (0.772) | 78 (78) | 88 (81) |
| SP-ALIGN^2)^ | 5 | 0.780 (0.744) | 72 (70) | 85 (84) |
| FNGUSHAK | 4 | 0.779 (0.777) | 75 (74) | 84 (86) |
| COFACTOR_HUMAN | 5 | 0.772 (0.768) | 81 (78) | 77 (78) |
| COFACTOR^2)^ | 5 | 0.772 (0.768) | 81 (78) | 77 (78) |
| 3DLIGANDSITE2 | 5 | 0.772 (0.763) | 75 (72) | 84 (85) |
| ATOME2_CBS^2)^ | 5 | 0.755 (0.722) | 72 (70) | 86 (79) |
| CONPRED-UCL^2)^ | 5 | 0.604 (0.524) | 56 (52) | 68 (61) |
| CHUO-BINDING-SITES | 5 | 0.467 (0.514) | 32 (39) | 86 (86) |
| BINDING_KIHARA^2)^ | 5 | 0.352 (0.368) | 60 (63) | 19 (27) |

1. Targets: T0652, T0697, T0721, T0737, T0744
2. Server predictors

Table S4. Comparison of different binding-site prediction methods on CAMEO ligand-binding site prediction targets with non-metal ligands in terms of median values (average in parentheses) of MCC, accuracy, and coverage

| **Servers** | **No. of common targets**^1)^ |  | | | **GalaxySite** | | |
| --- | --- | --- | --- | --- | --- | --- | --- |
|  |  | **MCC** | **Accuracy** | **Coverage** | **MCC** | **Accuracy** | **Coverage** |
| Naïve Homology^2)^ | 45 | 0.575 (0.500) | 100 (86) | 41 (34) | 0.801 (0.723) | 85 (77) | 78 (72) |
| Naïve Pocket^2)^ | 250 | 0.153 (0.148) | 50 (45) | 6 (7) | 0.646 (0.511) | 70 (57) | 61 (51) |
| Naïve Conservation^2)^ | 142 | 0.142 (0.149) | 17 (24) | 15 (22) | 0.656 (0.525) | 71 (60) | 61 (52) |
| FunFOLD^3)^ | 223 | 0.526 (0.471) | 69 (60) | 45 (44) | 0.672 (0.555) | 75 (62) | 64 (56) |
| HHfunc^4)^ | 110 | 0.631 (0.607) | 57 (53) | 89 (81) | 0.648 (0.501) | 78 (58) | 55 (49) |
| COACH^5)^ | 297 | 0.654 (0.585) | 86 (76) | 53 (51) | 0.652 (0.542) | 72 (61) | 61 (54) |

1. The numbers of predicted targets are different for different servers; thus, only common targets were considered for comparison.
2. <http://www.schwedelab.org/>
3. <http://www.reading.ac.uk/bioinf/FunFOLD/>, Roche, D.B., Tetchner, S.J. and McGuffin, L.J. (2011) FunFOLD: an improved automated method for the prediction of ligand binding residues using 3D models of proteins. *BMC bioinformatics*, **12**, 160.
4. <http://www.soeding.genzentrum.lmu.de/>, Fischer, J.D., Mayer, C.E. and Soding, J. (2008) Prediction of protein functional residues from sequence by probability density estimation. *Bioinformatics*, **24**, 613-620.
5. <http://zhanglab.ccmb.med.umich.edu/COACH/>, Yang, J., Roy, A. and Zhang, Y. (2013) Protein-ligand binding site recognition using complementary binding-specific substructure comparison and sequence profile alignment. *Bioinformatics*, **29**, 2588-2595.

**REFERENCES**

1. Kasahara, K., Kinoshita, K. and Takagi, T. (2010) Ligand-binding site prediction of proteins based on known fragment-fragment interactions. *Bioinformatics*, **26**, 1493-1499.

2. Huang, B. and Schroeder, M. (2006) LIGSITEcsc: predicting ligand binding sites using the Connolly surface and degree of conservation. *BMC structural biology*, **6**, 19.

3. Schmidt, T., Haas, J., Gallo Cassarino, T. and Schwede, T. (2011) Assessment of ligand-binding residue predictions in CASP9. *Proteins*, **79 Suppl 10**, 126-136.

4. Cassarino, T.G., Bordoli, L. and Schwede, T. (2013) Assessment of ligand binding site predictions in CASP10. *Proteins*.

5. Protein 3D structure prediction server CAMEO, Continuous Automated Model Evaluation; http://www.cameo3d.org/register.php?pid=registerServer&register_server_type=modelingServer.

6. Tripathi, A. and Kellogg, G.E. (2010) A novel and efficient tool for locating and characterizing protein cavities and binding sites. *Proteins*, **78**, 825-842.

7. Ngan, C.H., Hall, D.R., Zerbe, B., Grove, L.E., Kozakov, D. and Vajda, S. (2012) FTSite: high accuracy detection of ligand binding sites on unbound protein structures. *Bioinformatics*, **28**, 286-287.

1. ^*^ To whom correspondence should be addressed (myeong@amc.seoul.kr, chaok@snu.ac.kr) [↑](#footnote-ref-2)
